# Coadministration of Dexamethasone and *Melissa officinalis* Has Neuroprotective Effects in Rat Animal Model with Spinal Cord Injury 

**DOI:** 10.22074/cellj.2016.4868

**Published:** 2016-12-21

**Authors:** Seyed Ruhollah Hosseini, Gholamreza Kaka, Mohammad Taghi Joghataei, Mehdi Hooshmandi, Seyed Homayoon Sadraie, Kayvan Yaghoobi, Korosh Mansoori, Alireza Mohammadi

**Affiliations:** 1 Neuroscience Research Centre, Baqiyatallah University of Medical Sciences, Tehran, Iran; 2 Department of Anatomy, School of Medicine, Iran University of Medical Sciences, Tehran, Iran; 3 Neuroscience Research Centre, Shahid Beheshti University of Medical Sciences, Tehran, Iran; 4 Department of Anatomy, School of Medicine, Baqiyatallah University of Medical Sciences, Tehran, Iran; 5 Department of Physical Medicine and Rehabilitation, Iran University of Medical Sciences, Tehran, Iran

**Keywords:** Dexamethasone, *Melissa officinalis*, Neuroprotective, Spinal Cord Injury

## Abstract

**Objective:**

Spinal cord injury (SCI) causes inflammation, deformity and cell loss. It has been shown that *Melissa officinalis* (MO), as herbal medicine, and dexamethasone (DEX) are useful in the prevention of various neurological diseases. The present study evaluated combinational effects of DEX and MO on spinal cord injury.

**Materials and Methods:**

Thirty six adult male Wistar rats were used in this experimental study. The weight-drop contusion method was employed to induce spinal cord injury in rats. DEX and MO were administrated alone and together in different treatment groups. Intra-muscular injection of DEX (1 mg/kg) was started three hours after injury and continued once a day for seven days after injury. Intra-peritoneal (I.P) injection of MO (150 mg/ kg) was started one day after injury and continued once a day for 14 days.

**Results:**

Our results showed motor and sensory functions were improved significantly in the group received a combination of DEX and MO, compared to spinal cord injury group. Mean cavity area was decreased and loss of lower motor neurons and astrogliosis in the ventral horn of spinal cord was significantly prevented in the group received combination of DEX and *Melissa officinalis*, compared to spinal cord injury group. Furthermore, the findings showed a significant augmentation of electromyography (EMG) recruitment index, increase of myelin diameter, and up-regulation of myelin basic protein in the treated group with combination of DEX and MO.

**Conclusion:**

Results showed that combination of DEX and MO could be considered as a neuroprotective agent in spinal cord injury.

## Introduction

Spinal cord injury (SCI) causes severe damage to the function of motor and sensory neurons and may lead to paraplegia and tetraplegia (1). Injury and pathology of the spinal cord have generally poor prognosis. SCI pathophysiology is a biphasic process including primary and secondary steps. The primary process is associated with energy deprivation and physical deformation, whereas the secondary process includes cascades of cellular and biological processes which are mostly triggered by the primary stage (2). 

Following SCI, neurons respond to an initial period of growth, followed by growth cone collapse and failure of significant axon regeneration. Two major factors contributing to the inhibitory milieu of the injured central nervous system (CNS) are myelin associated proteins and glial scar (3). Regulation of both axonal degenerative and regenerative processes after injury is mediated by the inflammatory cascades (4).

Various forms of steroids have been used in the treatment and management of SCI for many years. Historically, the rational for application of corticosteroids in the management of neural trauma was extended from their role in decreasing edema in the management of brain tumors. Moreover, their anti-inflammatory properties were thought to be useful in alleviating the secondary injury pathophysiology of SCI. Steroid medication inhibits inflammatory response and consequently recruitment of macrophages (5). It has generally been accepted that systemic steroid enhances functional recovery after a crush injury to rat sciatic nerve (4).

Administration of methylprednisolone (MP) within the first few hours up to 24 hours after injury is the acute clinical treatment of spinal cord injured patients (6). MP is clinically used at high dose, as an anti-inflammatory agent to decrease the secondary process (7). However, the experimental as well as clinical data using MP after SCI remain largely inconclusive and controversial, with regard to the improved functional outcome (6). Dexamethasone (DEX) has a pharmacological profile and a chemical structure similar to MP (8), while it is a stable and more powerful substitute compared to MP (9).

*Melissa officinalis* (MO), commonly known as lemon balm (family: Lamiaceae), is one of the oldest and still most common medicinal plants. The MO leaves have been used conventionally to prepare tea, with the aim of calming and anti-spasmolytic effects. It has been reported that the most commonly known therapeutic properties of MO extract are sedative, anti-spasmodic, carminative, anti-bacterial, anti-viral, anti-inflammatory, anti-oxidant, as well as neuroprotective effects (10). Chemical constituents with anti-oxidative activity can be found at high concentrations in this plant, and can be responsible for its preventive effects in various degenerative conditions (11), such as ischemic brain injury (12) and Alzheimer disease (13). Furthermore, it has been shown that oral administration of MO results in cell proliferation and differentiation by decreasing serum corticosterone levels and also by increasing Gamma-Amino Butyric Acid (GABA) levels in the mouse dentate gyrus (14). Previously we showed that the effective dose of MO was 150 mg/kg in spinal cord injury contusive model. In addition, we determined that MO extract was effective to improve motor, sensory and cellular function after spinal cord injury (15)

Various therapeutic approaches are now accessible for SCI, but many of them are expensive and lead to various side effects (16). Furthermore, in recent years, application of corticosteroids has been controversial (17). Although anti- inflammatory and neuroprotective effects of DEX are powerful and multifactorial, there are a number of mechanisms of inflammation and neurodegeneration which is not affected by this drug dosage. In this study, it is hypothesized that combination of DEX and MO could play a role in preventing the harmful effects triggered by neural damage, and it can also promote neurological functions after SCI.

## Materials and Methods

### Animals

In this experimental study, after obtaining the approval of the Institutional Review Board of the University, all experiments were conducted in accordance with the Guidelines of the Animal Care and Use Ethics Committee of Baqiyatallah University of Medical Sciences (Iran). Thirty six adult male Wistar rats weighting 190-220 g (Razi Institute, Iran) were maintained under standard laboratory conditions. Animals were housed in an environment of 21 ± 2˚C with a relative humidity of 10 to 50% and a 12 hours light-dark cycle. Food and water were always available.

### Surgical procedure for spinal cord injury 

In order to make SCI, the animals were anesthetized with 80 mg/kg ketamine hydrochloride and 10 mg/ kg xylazine hydrochloride (Alfasan, Netherlands) intra-peritoneally (I.P). Weight-drop contusion method was conducted to induce SCI in rats. The skin and subcutaneous tissues in the thoracolumbar T12-L1 region were incised. After penetration of paravertebral muscle fascia, muscles were peeled laterally using blunt dissection forceps. The spinal cord segment at T12-L1 level was exposed by total laminectomy. The animals were subjected to an impact of 10 g weight (stainless steel rod, 3 mm diameter tip) dropped vertically in the center of the exposed spinal cord from the height of 25 mm. In sham group, all of the mentioned procedures were carried out, except the spinal cord contusion. Finally, the incision was sutured completely (18).

Core body temperature of animals was maintained at 36.5-37.5˚C during and after the procedures. The rats were treated with gentamicin (Caspian Tamin, Iran) twice a day for the first 3 days (40 mg/kg, intramuscular injection) as prophylaxis against urinary tract infection. The urinary bladders were pressed three times a day, as long as bladder function returned to normal. The rats were also injected subcutaneously with 25 ml/kg lactated Ringer’s (Caspian Tamin, Iran) for two days after SCI as once a day (19).

### Plant collection and extraction

The plant was obtained from the Institute of Medicinal Plants (Karaj, Iran). Dried leaves powder of MO was macerated at room temperature in 70% ethanol (1 g/10 ml) and extracted for a week. On day seven, the ethanolic extract was refined and evaporated under reduced pressure to remove the ethanol. The dry extract was suspended in the normal saline, thus alcoholic extract of MO was prepared (11, 15).

### Animal groups and drugs administration 

Rats were randomly divided into six groups as follows: group І: intact group (n=5), group ІІ: sham rats subjected to laminectomy without SCI (n=5), group ІІІ: rats subjected to laminectomy and SCI (n=5), group ІV: rats subjected to laminectomy, SCI and treated with 150 mg/kg MO (SCI-MO, n=7), group V: rats subjected to laminectomy, SCI and treated with 1 mg/kg DEX (SCI-DEX, n=7), group VІ: rats subjected to laminectomy, SCI and treated with 150 mg/kg MO+1 mg/kg DEX (SCI-DEX-MO, n=7). MO was daily injected I.P into the treated rat groups, starting one day after injury for 14 days. DEX was every day injected intra-muscularly (I.M) into the treated rat groups, starting three hours after injury, for seven days.

### Neurological examination 

For assessment of neurological function, Basso-Beattie-Bresnahan (BBB) scale was used for open-field motor testing in all rat groups. The BBB scale is a 21 point scale, ranging from zero to 21 (20), rating locomotion on aspects of hind limb function such as weight support, stepping ability, coordination and toe clearance (18). All functional scores were obtained on days 1, 7, 14, 21, 28, 35, 42, 49 and 56 by two individuals who were blinded on treatment. The final score of each animal was obtained from the mean value of both examiners.

Behavioral test for evaluating sense of pain were performed by means of hot-water test for the hind limbs after SCI (scores were obtained on days 1, 7, 14, 21, 28, 35, 42, 49 and 56). The response to heat stimulation was measured by the latency of hind limb paw withdrawal to hot-water of 50˚C. Both paws of rats were kept in a hot water container, respectively. For each rat six trials were obtained (three trial for each paw), and mean of these trials were recorded. In this experiment, non-responders were removed from the hot-water container after 60 seconds (21).

### Electrophysiological evaluations

Spontaneous rest activity was recorded from hind limb flexor muscle bilaterally. EMG recording was done by 23 gauge needles for 10 seconds one day prior to sacrificing animals. EMG signal was amplified (Grass, Astro-Med Inc., West Warwick, RI, USA), digitized (5 kHz, Digi-data 1322A, Axon instruments, Foster City, CA, USA) and filtered (30-300 Hz) (22). After recording, the recruitment index of motor units was acquired via compression of 10 seconds of recording to 1 second by EMG software. The recruitment index was scored on an ordinal scale (0 to ++++) (23). 

### Histology and immunohistochemistry

On day 57, all rats were anesthetized (100 mg/kg sodium pentobarbital; I.P). Thereafter, they were intra-cardially perfused with 0.9% saline, followed by 10% buffered formalin. Spinal cord segment at the level of T12-L1 was dissected, post-fixed in 10% buffered formalin overnight, cryoprotected in 30% sucrose for 48 hours and serially transverse-sectioned using a cryostat (B1155800 Sakura, Japan) at 10 µm thickness. All sections were processed for hematoxylin and eosin staining and assessed under light microscopy (18). Standard immunohistochemistry for the glial scar [glial fibrillary acidic protein (GFAP)] and myelination [myelin basic protein (*MBP*)] was performed for all of the sections. For immunohistochemistry, sections from formalin-fixed, paraffin embedded spinal cord tissues were dewaxed, rehydrated, and retrieval of antigens was performed. After incubation with 3% H_2_O_2_ in methanol, as well as normal non-immune goat serum, the sections were incubated with rabbit anti-active GFAP polyclonal antibody and mouse monoclonal *MBP* primary antibody (Santa Cruz Biotechnology, USA), at a dilution of 1:200 at 4˚C for overnight, followed by biotinylated goat anti-rabbit IgG for 20 minutes at room temperature, and subsequently incubated with streptavidin-peroxidase (All from Santa Cruz Biotechnology, USA). PBS was replaced to primary antibody as the negative control. DAB chromogen was applied for visualization of peroxidase activity. Finally, the sections were counterstained with hematoxylin (15, 24).

### Histomorphometric analysis

The lesion area, including the cavity and surrounding damaged tissue in area of 3562500 µm^2^was then measured by using an image analyzing software (Motic 2.1, Italy). In addition, the number of lower motor neurons in area of 5700 µm^2^as well as the number of positive GFAP astrocyte perikaryons in ventral horn, area of 35625 µm^2^, was measured. Only those cells that showed clearly discernible nucleus were counted. Densities of myelin, in dorsal white matter, and astrogliosis, in ventral horn of spinal cord, were evaluated using histolab software (Zist Rahe Danesh Co., Iran). Five sections from each case were evaluated, and mean values were obtained for each animal. Cell counting and densitometry analyses were carried out by two observers who were blind on the specific experimental conditions of the analyzed tissues on images acquired at ×40, ×400 and ×1000 magnifications (25).

### Transmission electron microscopic studies

For electron microscopy, spinal cords from five rats in each treatment group were processed into 3 mm^3^small blocks surrounding the injury epicenter. They were fixed for one hour in a mixture of glutaraldehyde (1.5%) and paraformaldehyde (3%), followed by washing three times in 0.1 M sodium cacodylate and 3 mM CaCl_2_. Samples were then post-fixed in potassium ferrocyanide (0.8%) and osmium tetroxide (1%) for one hour followed by three times washing in 0.1 M sodium cacodylate, and 3 mM CaCl_2_ (All from Sigma, USA). Upon a brief rinse with dH_2_O, samples were embedded in Eponate 12 (Pella), and cured at 60˚C for 2 days. Spinal cord sections (80 nm in thickness) corresponding to the site of the lesion were cut on a Riechert Ultracut E with a Diatome diamond knife, collected on formvar-coated 1×2 mm^2^copper grids, and stained with uranyl acetate followed by lead citrate. Sections were examined on a Hitachi 7600 transmission electron microscope (TEM) operating at 80 kV. The myelin index (MI) was measured by means of the ratio for axon diameter to axon diameter plus its myelin sheath (26, 27).

### RNA extraction and reverse transcription polymerase chain reaction 

T12-L1 segments of spinal cord from various groups were homogenized and total RNAs were isolated using RNeasy Mini Kit (Qiagen, USA) according to the manufacturer’s protocol. Approximately 1 µg total RNA from each sample was reverse transcribed into cDNA according to the manufacturer’s instructions using the iScripTM cDNA Synthesis Kit (Bio-Rad Laboratories, USA). Glyceraldehyde 3-phosphate dehydrogenase (GAPDH) was applied as internal control. We used the following sequences for the forward and reverse primers:

For *MBP*

F: 5´-CTCTGGCAAGGACTCACACA-3´

R: 5´-GTCTCTTCCTCCCCAGCTA-3´

For *GAPDH*

F: 5´-CCACCCATGGCAAATTCC-3´

R: 5´-CAGGAGGCATTGCTGATGAT-3'

The housekeeping gene, GAPDH, was used for normalization of *MBP* mRNA expression. Samples were subjected to 25-35 cycles at 95˚C for 30 seconds, 60˚C for 30 seconds, and 72˚C for 1 minute on GeneAmp PCR System 9700 (Perkin Elmer, USA) in 25 µl reaction volumes. After amplification, reverse transcription polymerase chain reaction (RT-PCR) products were separated on a 1% agarose gel containing 0.5 mg/ml ethidium bromide. The amplified cDNA fragments were visualized under ultraviolet light (28).

### Statistical analyses

Data obtained from motor and sensory functions at each time-point and electromyographic activity between different groups was analyzed using two-way analyses of variance (ANOVA). The histomorphometric, immunostaining data, densitometry and electron microscopic data were analyzed using ANOVA. In both tests, ANOVA was followed by Post Hoc Bonferroni’s multiple comparison tests using GraphPad Prism 6.0 (Graph-Pad Software, San Diego, CA). Data have been presented as the mean ± SEM. A significance level (P value) of 0.05 was predetermined for all statistical analyses.

## Results

In all experiments there was no significant difference between sham and intact groups. Moreover, significant differences have been determined between intact and SCI groups (P<0.001) in all experiments. In fact, the main index for SCI model induction was this significance.

### Neurological function results

#### Coadministration of dexamethasone and *Melissa officinalis* extracts increased motor function after spinal cord injury

**Fig.1:**
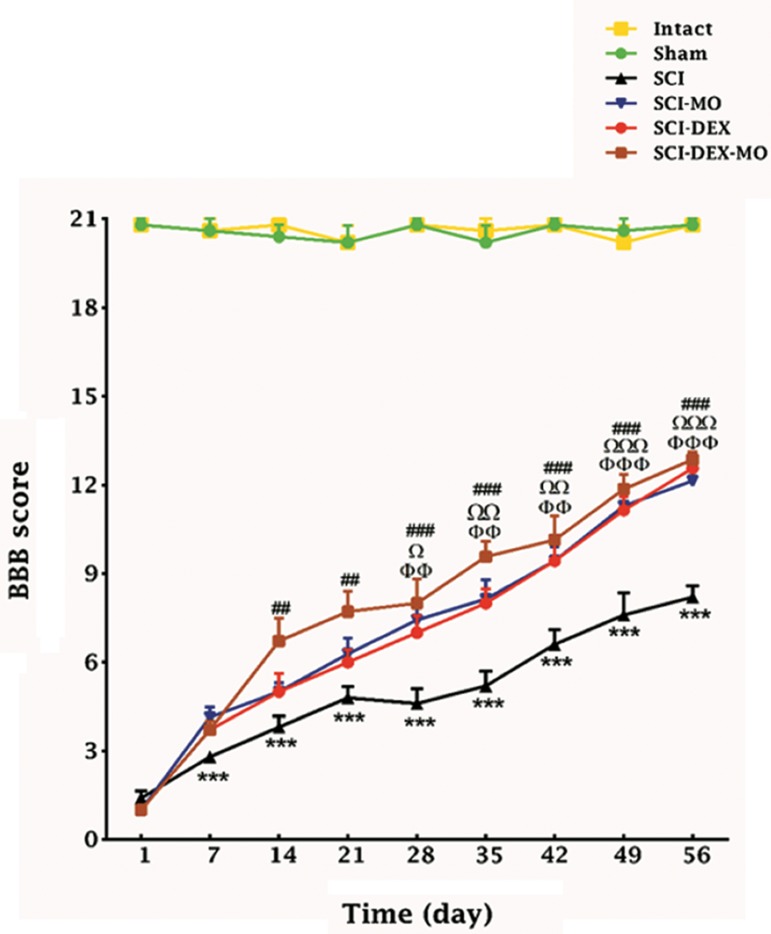
Effect of DEX-MO treatment on motor function after SCI. Intra-muscular injection of DEX (1 mg/kg) was started three hours after injury and continued once a day for 7 days after in- jury. I.P injection of MO (150 mg/kg) was started one day after injury and continued once a day for 14 days after injury. Data are represented as mean of BBB score ± SEM (n=5-7) and analyzed by two-way ANOVA followed by post-hoc Bonferroni’s multiple comparison test. ***; P<0.001 vs. intact, Ω, ΩΩ, ΩΩΩ; Significant difference be- tween SCI-DEX and SCI (P<0.05, P<0.01 and P<0.001 respective- ly), Φ, ΦΦ and ΦΦΦ; Significant difference between SCI-MO and SCI, #, ##, ###; Significant difference between SCI-DEX-MO and SCI (P<0.05, P<0.01 and P<0.001 respectively), DEX; Dexametha- sone, MO; *Melissa officinalis*, SCI; Spinal cord injury, I.P; Intra- peritoneal, and BBB; Basso-Beattie-Bresnahan.

While SCI caused immediate paraplegia (loss of hind limb movement), the SCI group showed significant changes in locomotion scores in comparison with intact group. DEX significantly improved locomotor function in rats as compared to SCI group. But when we added MO (150 mg/kg) I.P one day after injury, it significantly improved locomotor function in rats, compared to SCI and SCI-DEX groups. Application of two-way ANOVA showed significant interaction between variables, such as treatments and time [F (40, 270)=13.02, P<0.001]. Application of post-hoc Bonferroni’s multiple comparison tests revealed significant improvement in motor function following 150 mg/ kg MO treatment on days 28, 35 and 42 (P<0.0 01), 49 and 56 (P<0.001) and DEX therapy on days 28 (P<0. 05), 35, 42 (P<0. 01), 49 and 56 (P<0.001). Combination of DEX-MO improved motor function significantly on days 14, 21 (P<0.01), 28, 35, 42, 49 and 56 (P<0.001) (Fig.1).

#### Coadministration of dexamethasone and *Melissa officinalis* extract increased sensory function after spinal cord injury

**Fig.2:**
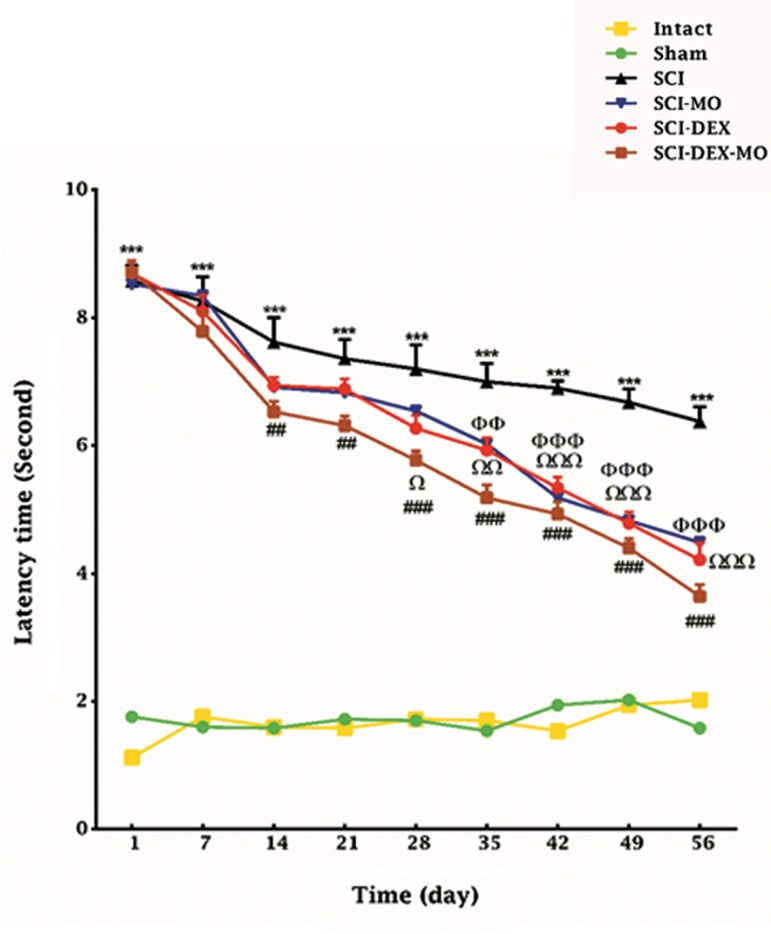
Effect of DEX-MO treatment on sensory function after SCI. Intra-muscular injection of DEX (1 mg/kg) was started three hours after injury and continued once a day for 7 days after in- jury. I.P injection of MO (150 mg/kg) was started one day after injury and continued once a day for 14 days after injury. Data are represented as mean of latency time ± SEM, (n=5-7) and analyzed by two-way ANOVA followed by post-hoc Bonferroni’s multiple comparison test. ***; P<0.001 vs. intact, Ω, ΩΩ, ΩΩΩ; Significant difference be- tween SCI-DEX and SCI (P<0.05, P<0.01 and P<0.001 respec- tively), Φ, ΦΦ and ΦΦΦ; Significant difference between SCI-MO and SCI, #, ##, ###; Significant difference between SCI-DEX-MO and SCI (P<0.05, P<0.01 and P<0.001 respectively), DEX; Dexa- methasone, MO; *Melissa officinalis*, SCI; Spinal cord injury, and I.P; Intra-peritoneal.

Statistical evaluations revealed that the mean of latency time for response to painful stimulus was significantly decreased in SCI- MO group versus SCI group. But when we added intramuscular DEX treatment three hours after injury, it significantly improved sensory recovery in rats, compared to SCI, SCI-MO and SCI-DEX groups. Application of two-way ANOVA showed significant interaction between variables including treatments and time [F (40, 270)=14.41, P<0.0001]. Application of post-hoc Bonferroni’s multiple comparison test revealed significant improvement in sensory function following MO injection in comparison with SCI group on days 35 (P<0.01), 42, 49 and 56 (P<0.001) post-injury. Treatment with DEX decreased latency time in comparison with SCI group on days 28 (P<0.05), 35 (P<0.01), 42, 49 and 56 (P<0.001) post-injury. When we combined DEX with MO, it significantly improved sensory function in rats compared to SCI, SCI-DEX and SCI-MO groups, separately. Application of post-hoc Bonferroni’s multiple comparison test revealed significant improvement in sensory function following DEX- MO treatment on days 14, 21 (P<0.01) 28, 35, 42, 49 and 56 (P<0.001, Fig.2). 

### Electrophysiological results

#### Coadministration of dexamethasone and *Melissa officinalis* extract increased recruitment pattern of hind limbs after spinal cord injury

**Fig.3:**
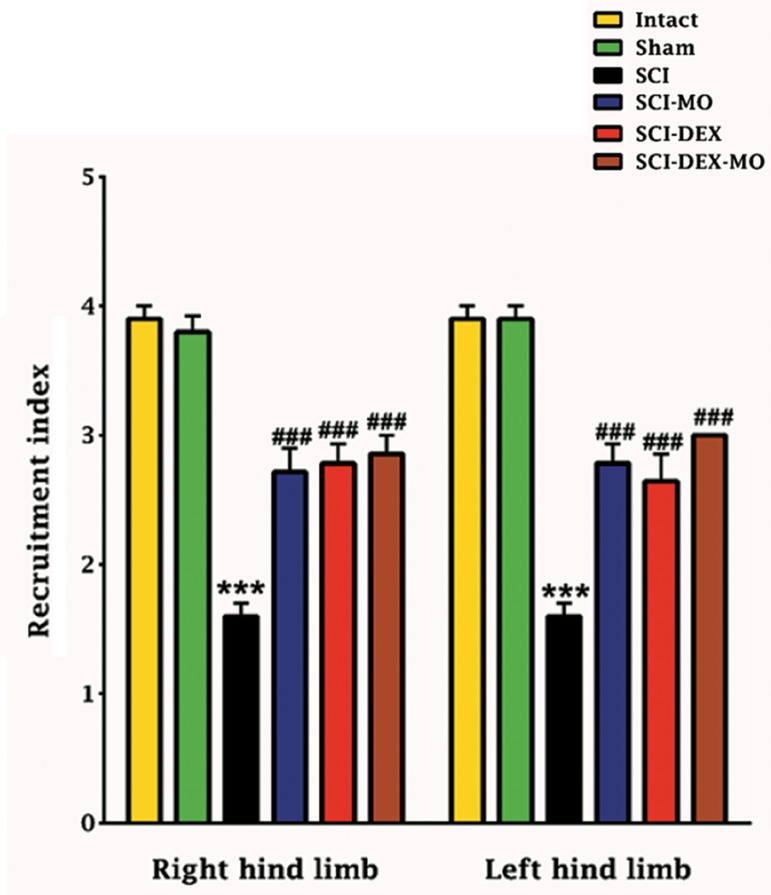
Effect of DEX-MO on electromyographic activity after SCI. Intra-muscular injection of DEX (1 mg/kg) was started three hours after injury and continued once a day for 7 days after in- jury. I.P injection of MO (150 mg/kg) was started one day after injury and continued once a day for 14 days after injury. Data are represented as mean of recruitment index ± SEM, (n=5-7) and analyzed by two-way ANOVA followed by post-hoc Bonferroni’s multiple comparison test. ***; P<0.001 vs. intact, ###; P<0.001 vs. spinal cord injury, DEX; Dexamethasone, MO; *Melissa officinalis*, SCI; Spinal cord injury, and I.P; Intra-peritoneal.

By application of two-way ANOVA, although no significant difference between right and left hind limb was determined, statistical analysis showed that the means of recruitment index were increased significantly for left and right hind limbs in SCI-MO, SCI-DEX and SCI-DEX-MO groups versus SCI group [F (5, 60)=60.27, P=0.0001]. Application of post-hoc Bonferroni’s multiple comparison test as well as Bartlett’s test for equal variances revealed significant improvement in electrophysiological activity of left and right hind limbs, following 150 mg/kg of MO extract administration, DEX therapy and combination of DEX-MO (P<0.001) in comparison with SCI group (Fig.3).

### Histological results

#### Combination of dexamethasone and *Melissa officinalis* extract reduced the cavity formation after spinal cord injury

**Fig.4:**
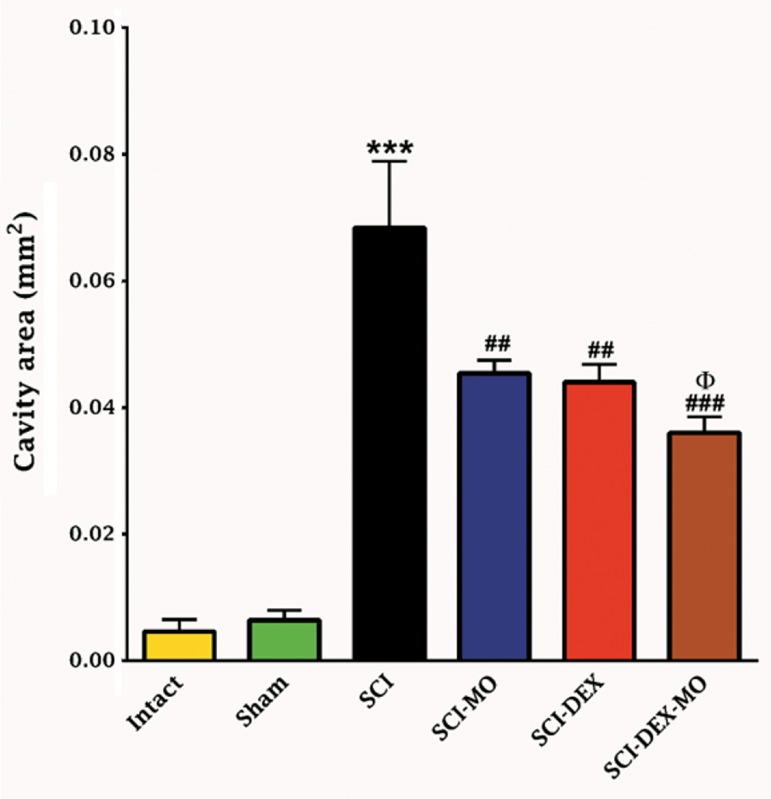
Effect of DEX-MO treatment on cavity formation after SCI. Intramuscular injection of DEX (1 mg/kg) was started three hours after injury and continued once a day for 7 days after injury. I.P injection of MO (150 mg/kg) was started one day after injury and continued once a day for 14 days after injury. Data are represented as mean of the cavity area ± SEM (n=5-7) and analyzed by one-way ANOVA followed by post-hoc Bonferroni’s multiple comparison test. ***; P<0.001 vs. intact, ##; P<0.01, ###; P<0.001 vs. spinal cord injury, Φ; Significant difference between SCI-DEX-MO and SCI- MO (P<0.05), DEX; Dexamethasone, MO; *Melissa officinalis*, SCI; Spinal cord injury, and I.P; Intra-peritoneal.

In the intact group, spinal cord segments were undamaged in both white and gray matter. Application of one-way ANOVA revealed that the mean of cavity size (mm^2^) was significantly reduced in treatment groups [F (5, 30)=30.17, P=0.0001]. Also post-hoc Bonferroni’s multiple comparison test illustrated significant decrease in the mean cavity area in SCI-MO, SCI-DEX (P<0.01) and SCI-DEX-MO (P<0.001) groups. Application of one-way ANOVA revealed that the mean of cavity size in SCI-DEX-MO group decreased significantly in comparison with SCI- MO group (P<0.05, Fig.4).

#### Coadministration of dexamethasone and *Melissa officinalis* extract prevented lessening of lower motor neurons in ventral horn of spinal cord after injury 

Statistical evaluations revealed significant differences between SCI-MO, SCI-DEX and SCI- DEX-MO groups versus SCI group in the number of ventral horn lower motor neurons [F (5, 30)=18.07, P=0.0001]. Application of post-hoc Bonferroni’s multiple comparison test as well as Bartlett’s test for equal variances revealed significant increase in the number of ventral horn motor neurons in SCI- MO (P<0.01), SCI-DEX (P<0.05) and SCI-DEX- MO (P<0.001) treatment groups, rather than SCI group. Application of one-way ANOVA revealed no significant difference between SCI-DEX-MO and SCI-MO groups (Figes.5, 6).

**Fig.5:**
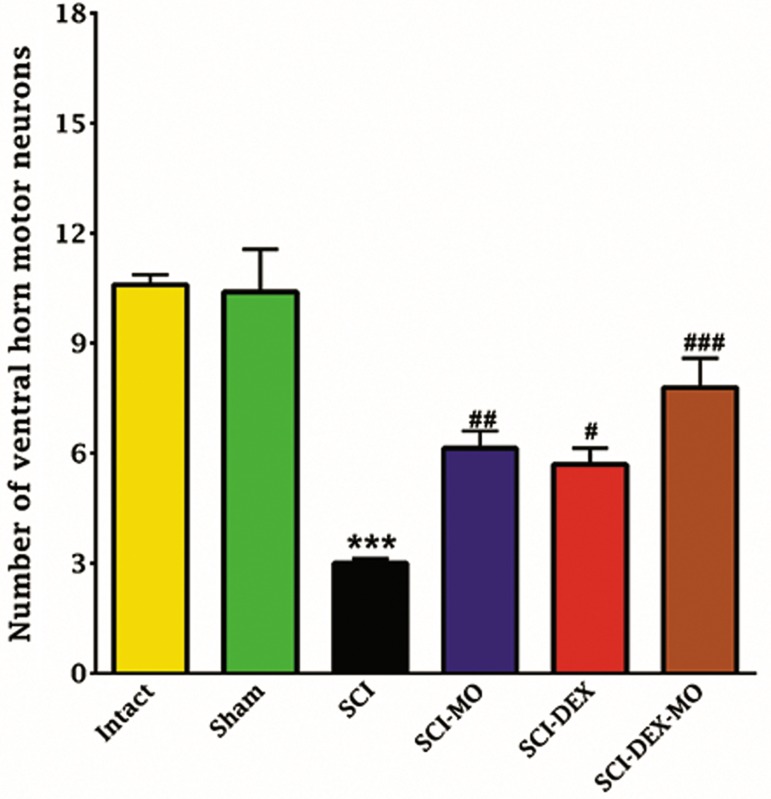
Effect of DEX-MO treatment on cell loss in ventral horn of spinal cord after injury. Intra-muscular injection of DEX (1 mg/kg) was started three hours after injury and continued once a day for 7 days after injury. I.P injection of MO (150 mg/kg) was started one day after injury and continued once a day for 14 days after injury. Data are represented as mean number of ventral horn motor neurons ± SEM (n=5-7) and analyzed by one-way ANOVA followed by post-hoc Bonferroni’s multiple comparison test. ***; P<0.001 vs. intact, #; P<0.05, ##; P<0.01, ###; P<0.001 vs. spinal cord injury, DEX; Dexamethasone, MO; *Melissa officinalis*, SCI; Spinal cord injury, and I.P; Intra-peritoneal.

**Fig.6:**
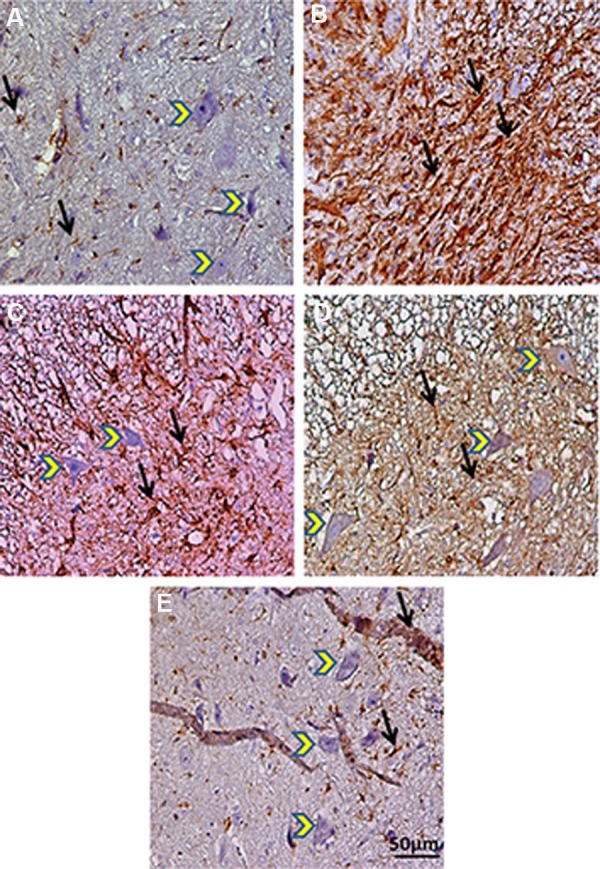
Transverse section of spinal cord showing the ventral horn gray matter of spinal cord at the level of T12-L1 of all groups evaluated on day 56. Black arrows illustrate glial fibrillary acidic protein (GFAP) astrocytes. Yellow arrows show lower motor neurons. A. Intact, B. SCI, C. SCI-DEX, D. SCI-MO, and E. SCI-DEX-MO. SCI; Spinal cord injury, DEX; Dexamethasone, and MO; *Melissa officinalis*.

### Immunohistochemistry and transmission electron microscope results

#### Coadministration of dexamethasone and *Melissa officinalis* extract decreased GFAP expression after spinal cord injury 

Statistical evaluations showed that number of GFAP^+^ astrocytes were significantly increased in SCI group, however, this activation was significantly attenuated in the treatment groups [F (5, 30)=48.23, P<0.0001]. Application of post- hoc Bonferroni’s multiple comparison test as well as Bartlett’s test for equal variances revealed significant decrease in the GFAP expression in SCI-MO, SCI-DEX (P<0.01) and SCI-DEX-MO (P<0.001) treatment groups versus SCI group. In addition, Application of one-way ANOVA revealed significant difference between SCI-DEX-MO and SCI-MO groups (P<0.01) (Figes.6, 7).

**Fig.7:**
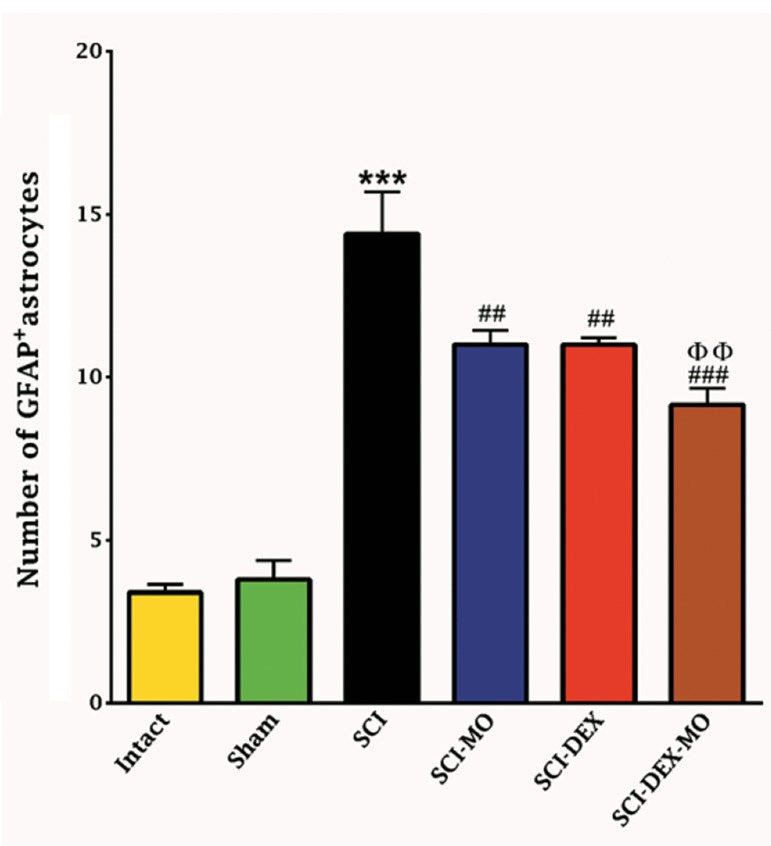
Effect of DEX-MO treatment on astrogliosis formation in ventral horn of spinal cord after injury. Intra-muscular injection of DEX (1 mg/kg) was started three hours after injury and con- tinued once a day for 7 days after injury. I.P injection of MO (150 mg/kg) was started one day after injury and continued once a day for 14 days after injury. Data are represented as mean of GFAP^+^ astrocytes ± SEM (n=5-7) and analyzed by one-way ANO- VA followed by post-hoc Bonferroni’s multiple comparison test. ***; P<0.001 vs. intact, ##; P<0.01, ###; P<0.001 vs. spinal cord injury, ΦΦ; Significant difference between SCI-DEX-MO and SCI- MO (P<0.01), DEX; Dexamethasone, MO; *Melissa officinalis*, SCI; Spinal cord injury, I.P; Intra-peritoneal, and GFAP; Glial fibrillary acidic protein.

**Fig.8:**
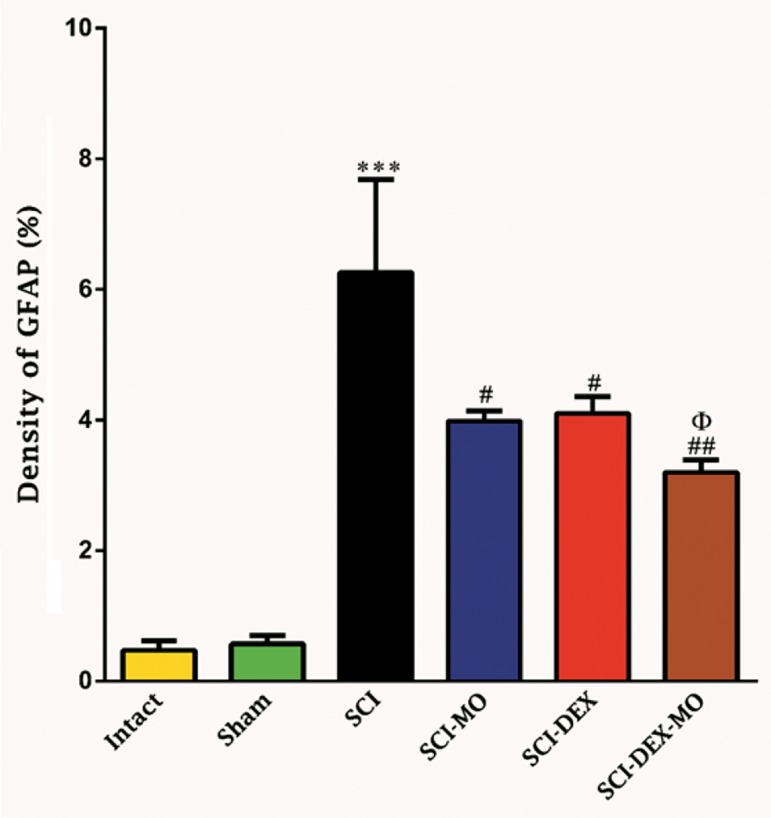
Effect of DEX-MO treatment on density of astrogliosis in ventral horn of spinal cord after injury. Intra-muscular injection of DEX (1mg/kg) was started three hours after injury and continued once a day for 7 days after injury. I.P injection of MO (150mg/kg) was started one day after injury and continued once a day for 14 days after injury. Data are represented as mean of gliosis density ± SEM, (n=5-7) and analyzed by one-way ANOVA followed by post-hoc Bonferroni’s multiple comparison test. ***; P<0.001 vs. intact, #; P<0.05, ##; P<0.01, Φ; Significant difference between SCI-DEX-MO and SCI-MO (P<0.05), DEX; Dexamethasone, MO; *Melissa officinalis*, SCI; Spinal cord injury, I.P; Intra-peritoneal, and GFAP; Glial fibrillary acidic protein.

Our data revealed that density of astrogliosis in the ventral horn of spinal cord was decreased significantly in the treated groups compared to SCI group [F (5, 30)=16.68, (P<0.001)]. Application of post-hoc Bonferroni’s multiple comparison test revealed significant decrease in density of gliosis in SCI-MO, SCI- DEX (P<0.05) and SCI-DEX-MO (P<0.01) treated groups versus SCI group. In addition, application of one-way ANOVA revealed significant difference between SCI-DEX-MO and SCI-MO groups (P<0.05) (Fig.8).

#### Coadministration of dexamethasone and *Melissa officinalis* extract enhanced remyelination after spinal cord injury

Application of one-way ANOVA showed that density of myelin in dorsal white matter of spinal cord was significantly increased in the treated groups versus SCI group [F (5, 30)=141.1, P=0.0001]. In addition, post-hoc Bonferroni’s multiple comparison test revealed that density of myelin was significantly increased in the SCI-MO, SCI-DEX (P<0.05) and SCI-DEX-MO (P<0.001) groups, compared to SCI group. Application of one-way ANOVA revealed significant difference between SCI-DEX-MO and SCI-MO groups (P<0.05, Figes.9, 10).

On the other hand, evaluation of electron microscopic pictures from all groups, using one- way ANOVA, showed that myelin index was decreased in the treated groups [F (5, 6)=128.5, P=0.0001]. In addition, post-hoc Bonferroni’s multiple comparison test revealed that myelin index was significantly decreased in the SCI-MO, SCI-DEX (P<0.01) and SCI-DEX-MO (P<0.001) groups, rather than SCI group. Application of one-way ANOVA revealed significant difference between SCI-DEX-MO and SCI-MO groups (P<0.05, Figes.10, 11).

### Reverse transcription polymerase chain reaction results

#### Dexamethasone in combination with *Melissa officinalis* extract enhanced expression of *MBP* after spinal cord injury 

To further confirm myelination process and synthesis of myelin basic protein by DEX-MO treatment after SCI, we used RT-PCR analysis. Changes in the level of mRNA after SCI were identified using standardized RT-PCR analysis. Qualitative analysis of RT-PCR finding in all groups showed considerable up-regulation of mRNA gene for *MBP* in the treated SCI-DEX-MO group compared to SCI group. Application of one-way ANOVA showed that density of RT-PCR bands was increased in the treated groups [F (5, 6)=946.7, P=0.0001]. In addition, post-hoc Bonferroni’s multiple comparison test revealed that density of RT-PCR bands was significantly increased in the SCI-MO (P<.001) groups, rather than SCI group. Application of one-way ANOVA revealed significant difference between SCI-DEX-MO and SCI-MO groups (P<0.05, Figes.12, 13). 

**Fig.9:**
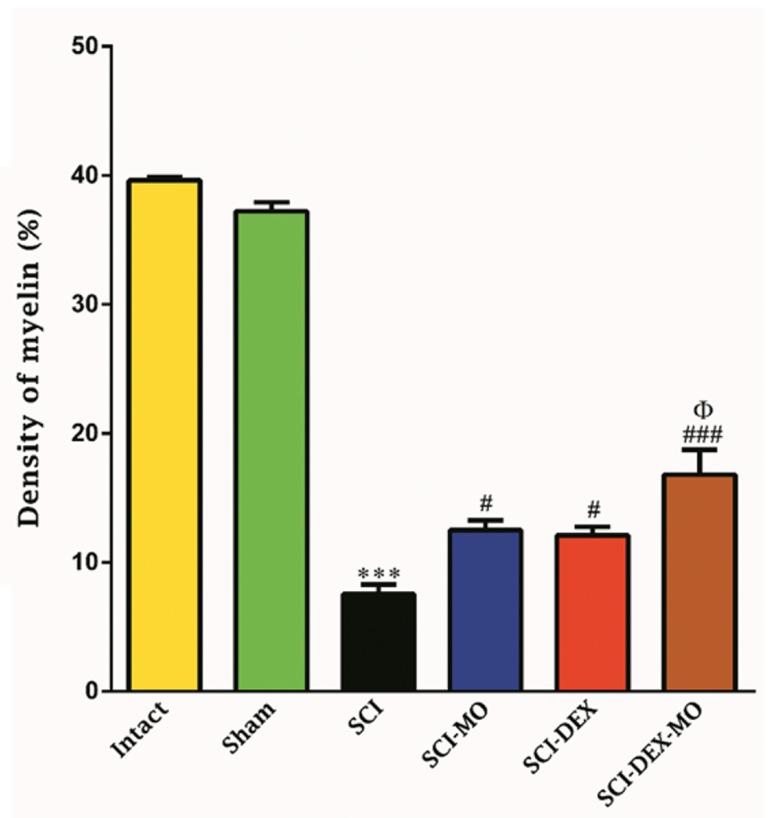
Effect of DEX-MO treatment on density of myelin in dorsal white matter of spinal cord after injury. Intra-muscular injection of DEX (1 mg/kg) was started three hours after injury and continued once a day for 7 days after injury. I.P injection of MO (150 mg/kg) was started one day after injury and continued once a day for 14 days after injury. Data are represented as mean of myelin density ± SEM, (n=5-7) and analyzed by one-way ANOVA followed by post-hoc Bonferroni’s multiple comparison test. ***; P<0.001 vs. intact, #; P<0.05, ###; P<0.001 vs. spinal cord injury, Φ; Significant difference between SCI-DEX-MO and SCI-MO (P<0.05), DEX; Dexamethasone, MO; *Melissa officinalis*, SCI; Spinal cord injury, and I.P; Intra-peritoneal.

**Fig.10:**
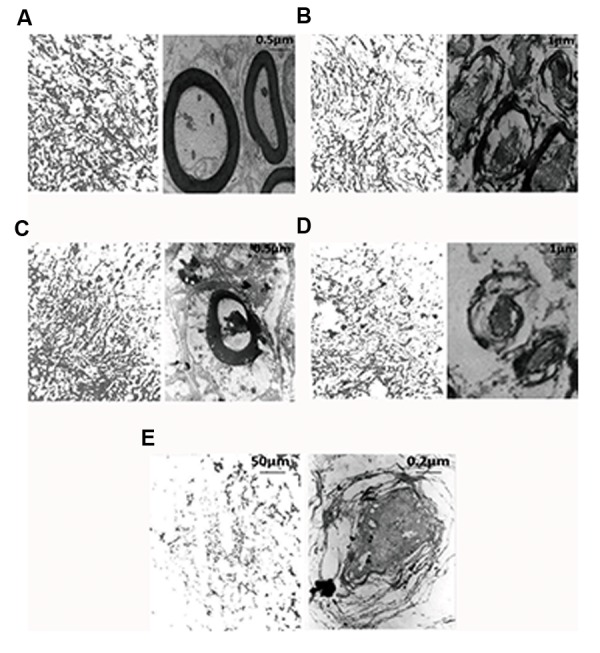
Ultra-structural characteristics of myelination in dorsal white matter of spinal cord at the level of T12-L1 of all groups evaluated on day 56. Low power view (left picture) reveals the distribution of myelinated axons. High power photographs (right picture) show the typical appearance of myelinated axons with extensive myelin sheath wrapped around an axon. Densitometry of MBP in dorsal white matter of spinal cord at the level of T12-L1 is shown in the left part of any electron microscopy pictures. A. Intact, B. SCI-MO, C. SCI-DEX- MO, D. SCI-DEX, and E. SCI.v DEX; Dexamethasone, MO; *Melissa officinalis*, and SCI; Spinal cord injury.

**Fig.11:**
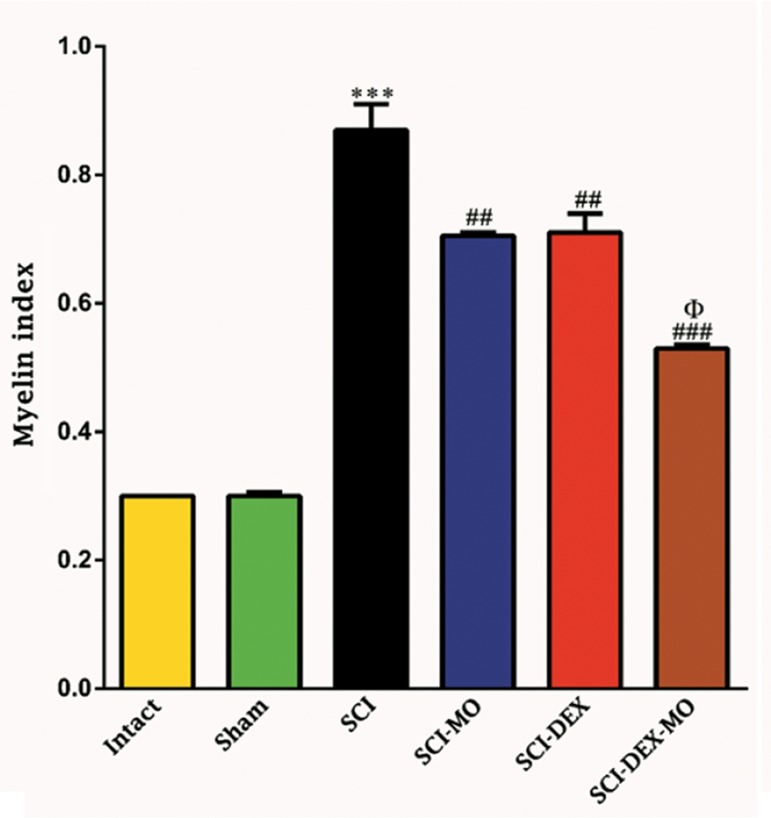
Effect of DEX-MO treatment on decreasing myelin index in dorsal white matter of the spinal cord after injury. Intra-muscular injec- tion of DEX (1 mg/kg) was started three hours after injury and continued once a day for 7 days after injury. I.P injection of MO (150 mg/ kg) was started one day after injury and continued once a day for 14 days after injury. Data are represented as mean of myelin index ± SEM (n=2) and analyzed by one-way ANOVA followed by post-hoc Bonferroni’s multiple comparison test. ***; P<0.001 vs. intact, ##; P<0.01, ###; P<0.001 vs. spinal cord injury, Φ; Significant difference between SCI-DEX-MO and SCI-MO (P<0.05), DEX; Dexamethasone, MO; *Melissa officinalis*, SCI; Spinal cord injury, and I.P; Intra-peritoneal.

**Fig.12:**
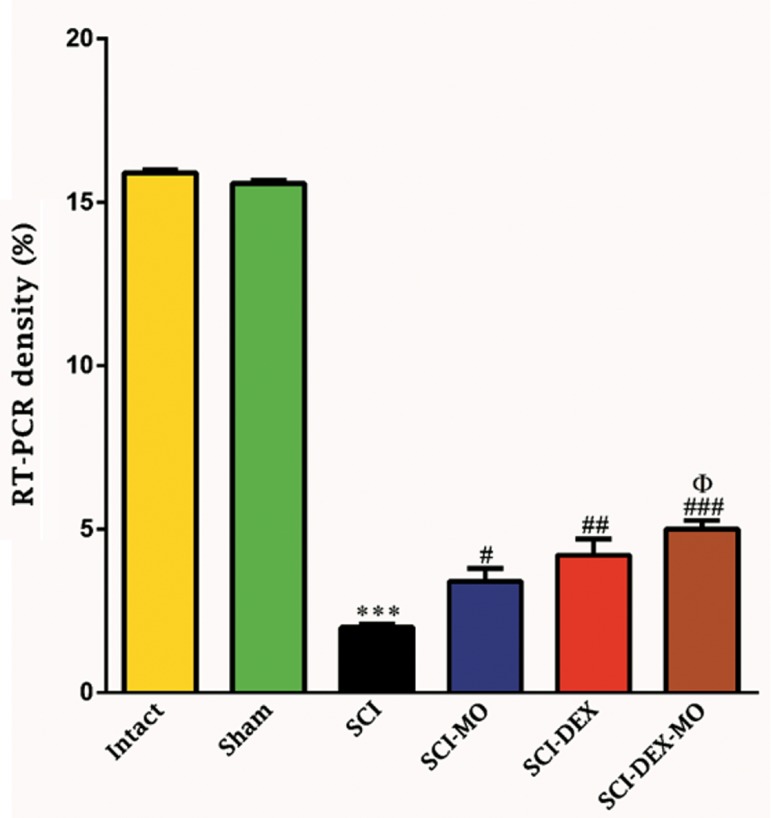
Effect of DEX-MO treatment on up-regulation of myelin basic protein in the spinal cord after injury. Intra-muscular injection of DEX (1 mg/kg) was started three hours after injury and continued once a day for 7 days after injury. I.P injection of MO (150 mg/kg) was started one day after injury and continued once a day for 14 days after injury. Data are represented as mean of RT-PCR bands density ± SEM (n = 2) and analyzed by one-way ANOVA followed by post-hoc Bonferroni’s multiple comparison test. ***; P<0.001 vs. intact. #; P<0.05, ##; P<0.01, ###; P<0.001 vs. spinal cord injury, Φ; Significant difference between SCI-DEX-MO and SCI- MO (P<0.05), DEX; Dexamethasone, MO; *Melissa officinalis*, SCI; Spinal cord injury, I.P; Intra-peritoneal, and RT-PCR; Reverse transcrip- tion-polymerase chain reaction.

**Fig.13:**
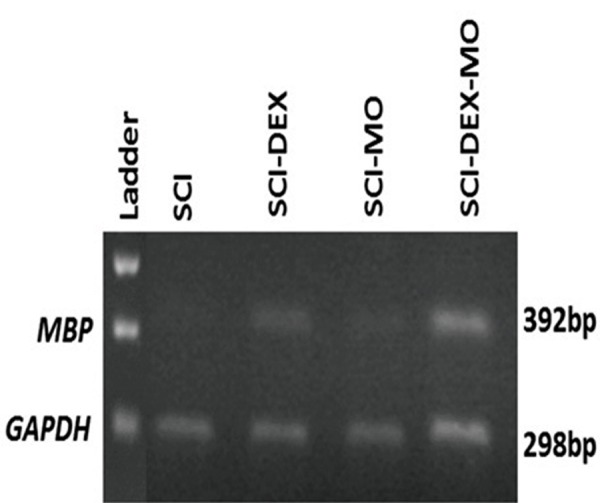
Expression of myelin basic protein (*MBP*) in injured and treated spinal cords of rats. RT-PCR analysis of myelin basic pro- teins depicting SCI, SCI-DEX, SCI-MO and SCI-DEX-MO groups. GAPDH, housekeeping gene, was used as loading control. DEX; Dexamethasone, MO; *Melissa officinalis*, SCI; Spinal cord injury, and RT-PCR; Reverse transcription-polymerase chain reaction.

## Discussion

The aim of present study was to investigate therapeutic effects of MO and dexamethasone combination in spinal cord injury. In this study, weight-drop contusion method was used to make SCI at T12-L1 level of spinal cord. This process causes gliosis, connective tissue deposition, demyelination, and cysts formation (25). Our Findings based on loosening of tissue and formation of cavity in gray and white matter was in line with previously studies (28, 29). We also showed that, within one day after SCI, the rats were paraplegic and unable to walk on the hind limb.

DEX and MO have therapeutic properties in SCI. Because neither of these drugs could individually inhibit disease, the present study investigated the effects of simultaneous administration of DEX and MO on SCI. The large number of inflammatory mediators are exists after SCI, that play an important role in the secondary process. So, blocking these mediators in the inflammatory cascade is crucial in achieving disease remission. In this study, we demonstrated that DEX (1 mg/kg) and MO (150 mg/kg) potentially reduced SCI when it was administered as a single treatment. Moreover, our results demonstrated that combinational therapy of DEX and MO could promote motor and sensory functions as well as recruitment of motor units in spinal cord and locomotors recovery in comparison with SCI group, at all time-points. The mechanism of the synergism observed with DEX and MO is not entirely clear. MO has an acetilcolinestrase inhibitory property (30), anti-cholinesterase increases the residence time of acetylcholine in the synapse. So, this allows rebinding of transmitter to nicotinic receptors (31). Moreover, DEX administration in postnatal rats enhances synthesis of acetylcholine in superior ganglia and promotes development of neonatal brain cholinergic nerve terminals. So, DEX may influence maturation of cholinergic neurons during ontogeny and has neuroprotective properties.

The present results showed that combination of MO and DEX can reduce cell loss as well as scar formation, improving sensory and motor functions. One possible explanation for this is that MO has anti-inflammation property, which has also been observed in the previous study. Anti- inflammatory effects of MO are due to rosmarinic acid, flavonoids and terpenoids presented in the extract. Probably flavonoids have more effective role by facilitating prostaglandin synthesis (32). Investigations have revealed that MO extracts have neuroprotective properties on ischemic damage mediated by the inhibition of oxidative stress, followed by the inhibition of apoptosis (12). Therefore, alleviating oxidative stress may be an effective way for treatment of SCI. MO has influential anti-oxidant effects and these effects more likely are exerted through two substances, including rosmarinic acid and benzodioxole, which are present in the extract. In addition, some compounds like acid linoleic acid and carnosic acid are also present in the extract, all of which have anti-oxidant properties (30). Moreover, DEX is powerful immunosuppressive and anti- inflammatory agent which is used therapeutically in several inflammatory pathologies. Findings have been shown that DEX improves recovery of neurological function in patients with SCI. It has been suggested that its primary mechanism of action inhibits secondary process after injury. This mechanism include inhibition of inflammatory responses, inhibition of free radical-induced lipid peroxidation, inhibition of synthesis of cytokines such as interlukin-1, interlukin-6 and TNF-α, reduction of intracellular calcium accumulation, improvement of energy metabolism and blood flow, reduced glutamate toxicity as well as degradation. In addition, DEX can increase synthesis of various neurotropic factors after injury. These trophic factors play an important role in cell survival after injury.

Findings also showed that combination of MO and DEX significantly decreased GFAP^+^astrocytes in comparison with SCI group. Reactive astrogliosis is a cellular response associated with injury of the nerve system. Activation of astrocytes and precursor cells play pro-inflammatory role in the lesion site that form barrier to axonal regeneration. As a result, inhibition of astrogliosis formation can help to promote axonal regeneration and neurological functions after SCI. Decreasing GFAP^+^astrocytes may be related to inhibition of pro-inflammatory cytokines and reactive oxygen species (ROS) by MO and DEX, when these two factors are key mediators of reactive astrogliosis in SCI (25). So, MO extract in combination with DEX can significantly promote motor and sensory functions. Generally, protection against progression of secondary injury to spinal cord neurons appears to be one of the most effective therapeutic strategies in limiting tissue injury and improving outcome of spinal cord trauma.

## Conclusion

SCI causes motor and sensory dysfunction, tissue deformity, cell death, formation of astrogliosis and degeneration of axons. In conclusion, combination of DEX and MO extracts improved motor and sensory dysfunction as well as promoting morphological improvement in spinal cord injury contusion model compared to SCI. Our results revealed that DEX can promote neuroprotective effects of MO, while further studies are needed to clarify the underlying mechanisms of these results. 
